# Confirming the Presence of Neurapraxia and Its Potential for Immediate Reversal by Novel Diagnostic and Therapeutic Ultrasound-Guided Hydrodissection Using 5% Dextrose in Water Without Local Anesthetics: Application in a Case of Acute Radial Nerve Palsy

**DOI:** 10.3390/diagnostics15151880

**Published:** 2025-07-26

**Authors:** Ho Won Lee, Jihyo Hwang, Chanwool Park, Minjae Lee, Yonghyun Yoon, Yeui-Seok Seo, Hyemi Yu, Rowook Park, Jaehyun Shim, Junhyuk Ann, Daniel Chiung-Jui Su, Teinny Suryadi, Keneath Dean Reeves, King Hei Stanley Lam

**Affiliations:** 1 Department of Orthopedic Surgery, Hallym University Gangnam Sacred Heart Hospital, 1 Singil-ro, Yeongdeungpo-gu, Seoul 07441, Republic of Korea; lhwghm@gmail.com (H.W.L.); hwangjihyo36@gmail.com (J.H.); 2 IncheonTerminal Orthopedics, Inha-ro 489beon-gil, Namdong-gu, Incheon 21574, Republic of Korea; humanpcw94@gmail.com (C.P.); mjlee951224@gmail.com (M.L.); ulsanpm@gmail.com (J.A.); 3 International Association of Regenerative Medicine, Namdong-gu, Incheon 21574, Republic of Korea; 4 MSKUS, 1035 E. Vista Way #128, Vista, CA 92084, USA; 5 The Board of Clinical Research, The International Association of Musculoskeletal Medicine, Kowloon, Hong Kong; 6 Department of Anatomy, Catholic Institute for Applied Anatomy, College of Medicine, Seoul 06591, Republic of Korea; prssys@area88ps.com; 7 Department of Plastic Surgery, Bio Plastic Surgery Clinic, Seoul 03186, Republic of Korea; myangel315@naver.com; 8 Department of Rehabilitation Medicine, Sae Yonsei Rehabilitation Clinic, Seoul 03186, Republic of Korea; prwook@naver.com; 9 Department of Neurosurgery, Chungdammadi Neurosurgery Clinic, Seoul 03186, Republic of Korea; jhyunshim@gmail.com; 10 Department of Physical Medicine and Rehabilitation, Chi Mei Medical Center, Tainan 710, Taiwan; dr.daniel@gmail.com; 11 Tempo Regeneration Center for Musicians, Tainan 700, Taiwan; 12 Physical Medicine and Rehabilitation, Synergy Clinic, Jakarta 11510, Indonesia; painfreedoc22@gmail.com; 13 Department of Physical Medicine and Rehabilitation, Hermina Podomoro Hospital, Jakarta 14350, Indonesia; 14 Rehabilitation Medicine, Private Practice, Kansas City, MO 64132, USA; deanreevesmd@gmail.com; 15 Faculty of Medicine, The University of Hong Kong, Hong Kong; 16 Faculty of Medicine, The Chinese University of Hong Kong, New Territory, Hong Kong; 17 The Board of Clinical Research, The Hong Kong Institute of Musculoskeletal Medicine, Kowloon, Hong Kong

**Keywords:** MSK ultrasound, point-of-care-ultrasound, acute radial nerve palsy, 5% dextrose in water without local anesthetic, spiral groove of humerus, compression neuropathy, peripheral nerve entrapment, Saturday night palsy, ultrasound-guided hydrodissection, wrist drop

## Abstract

**Background and Clinical Significance:** Radial nerve palsy typically presents as wrist drop due to nerve compression, with conventional management often yielding prolonged recovery. We report a case where ultrasound-guided hydrodissection (HD) with 5% dextrose in water (D5W) achieved immediate functional restoration, suggesting neurapraxia as the underlying pathology. **Case Presentation:** A 54-year-old diabetic female presented with acute left wrist drop without trauma. Examination confirmed radial nerve palsy (MRC grade 0 wrist extension), while radiographs ruled out structural causes. Ultrasound revealed fascicular swelling at the spiral groove. Under real-time guidance, 50 mL D5W (no local anesthetic) was injected to hydrodissect the radial nerve. Immediate post-procedure assessment showed restored wrist extension (medical research council (MRC) grade 4+). At one- and three-month follow-ups, the patient maintained complete resolution of symptoms and normal function. **Conclusions:** This case highlights two key findings: (1) HD with D5W can serve as both a diagnostic tool (confirming reversible neurapraxia through immediate response) and therapeutic intervention, and (2) early HD may circumvent prolonged disability associated with conservative management. The absence of electrodiagnostic studies limits objective severity assessment, though ultrasound localized the lesion. While promising, these observations require validation through controlled trials comparing HD to standard care, particularly in diabetic patients with heightened compression susceptibility. Technical considerations—including optimal injectate volume and the role of adjuvant therapies—warrant further investigation. US-guided HD with D5W emerges as a minimally invasive, surgery-sparing option for acute compressive radial neuropathies, with potential to redefine treatment paradigms when applied at symptom onset.

## 1. Introduction

Radial nerve palsy is a peripheral neuropathy characterized by motor deficits, primarily manifesting as wrist drop and weakness of finger extension, often accompanied by sensory disturbances over the dorsum of the hand. The most common pathophysiology involves neurapraxia secondary to external compression of the radial nerve, typically occurring during prolonged immobilization or mechanical pressure [1,2].

Compression leads to mechanical deformation, impaired microvascular perfusion, and subsequent demyelination, which contribute to the clinical manifestations of nerve dysfunction [2]. Although many cases recover spontaneously with conservative management, the extended duration of functional impairment—such as wrist drop—can result in temporary disability, decreased quality of life, and significant socioeconomic burden, particularly in modern working populations.

An important anatomical factor contributing to the vulnerability of the radial nerve is its course through the spiral groove of the humerus. The spiral groove is a well-known anatomical depression on the posterior aspect of the humerus through which the radial nerve and deep brachial artery pass [3]. Due to its superficial location and confinement between muscular and bony structures, the radial nerve at this site is particularly prone to compression injuries, explaining the frequent occurrence of conditions such as Saturday night palsy. Typical mechanisms of compression at the spiral groove include direct pressure from leaning the arm against a hard surface for extended periods (e.g., during sleep or prolonged immobilization), compression from external forces or tight clothing, or, less commonly, compression due to humeral fractures or masses [4].

In response to the limited effectiveness and prolonged recovery associated with conservative treatment of radial nerve palsy, ultrasound (US)-guided hydrodissection (HD) has emerged as a promising minimally invasive therapeutic option. According to Gragossian et al. (2025), patients typically recover 4 months after starting treatment, provided the nerve is not lacerated or torn, highlighting the slow course of spontaneous recovery [5]. Ultrasonography has been identified as the preferred modality for interventions around the spiral groove, and HD of the radial nerve near this region is a technique used to separate the nerve from surrounding tissues under ultrasound guidance by injecting a fluid (usually saline, sometimes with anesthetics or corticosteroids) [6,7,8,9]. This technique enables real-time visualization and mechanical decompression of the nerve [7]. Gill et al. (2022) reported that US-guided HD provides complete symptom resolution in radial tunnel syndrome, suggesting that this technique may offer a safe and effective alternative to surgical intervention in select cases of compressive neuropathy [9]. Dextrose 5% in water (D5W), when used as the primary injectate, appears to contribute additional therapeutic benefit to that of a mechanical hydrodissection effect alone, outperforming hydrodissection with saline or triemcinollone in the treatment of carpal tunnel syndrome [10,11,12,13,14].

When a patient presents with acute weakness due to peripheral nerve involvement, the key diagnostic challenge is determining whether the nerve dysfunction stems from neurapraxia, axonotmesis, or neurotmesis. Conventional teaching holds that immediate examination or early electromyography cannot reliably differentiate between these three distinct levels of nerve damage due to the presence of a conduction block. We describe a case of radial nerve palsy, likely caused by compression at the spiral groove, which was effectively treated with ultrasound-guided hydrodissection (HD) using 5% dextrose in water (D5W) without local anesthetics. The results suggest that acute weakness following apparent peripheral nerve injury may allow clinicians to confirm neurapraxia as the underlying pathology and assess its potential for rapid reversal. This approach could reduce or prevent lost work time, modify formal rehabilitation plans, and improve long-term outcomes.

This case report illustrates that ultrasound-guided dextrose hydrodissection can function as both a diagnostic and therapeutic intervention for acute compressive radial neuropathy. By providing immediate functional recovery, it helps confirm neurapraxia as the primary pathology, offering valuable clinical insights.

## 2. Case Presentation

A 54-year-old Korean woman presented with a sudden onset of left wrist drop that developed earlier that morning. On physical examination, the patient was unable to actively extend her left wrist (Supplementary Figure S1 and Supplementary Video S1).

The patient had a history of poorly controlled diabetes mellitus, managed with insulin therapy. No other underlying medical conditions were reported. The patient denied any previous history of similar symptoms or recent trauma. Plain radiographs of the upper extremity revealed no bony deformities or other structural abnormalities that could account for the neurological deficits (Supplementary Material Figure S2A,B).

Neurological examination, assessed using the Medical Research Council (MRC) scale, showed grade 0 wrist extension but preserved strength (grade IV or better) in shoulder abductors, elbow flexors, and extensors. Sensory testing revealed no paresthesia or hypoesthesia in the affected limb.

Pre-procedural ultrasound evaluation revealed several important findings. First, we observed epineural swelling without evidence of significant perineural fibrosis or vascular compression. Second, while the fascicular architecture remained intact, localized hypoechoic changes in select fascicles suggested the presence of focal edema. Most notably, during dynamic assessment of the spiral groove—performed while passively flexing and extending the patient’s wrist—we documented reduced nerve mobility relative to surrounding tissues. Although the overall cross-sectional area of the nerve remained within normal limits compared to the contralateral side, this combination of findings strongly suggested compressive neuropathy. The immediate functional restoration following hydrodissection further supports our interpretation that these ultrasound findings were consistent with acute neurapraxia at this anatomical site.

Given the clear clinical findings suggestive of radial nerve palsy, particularly in the context of poorly controlled diabetes as a potential predisposing factor, US-guided HD with D5W without local anesthetics (LAs) was recommended. This decision followed a thorough discussion of other conservative treatment options, and the patient provided consent for the procedure.

**Figure 1:**
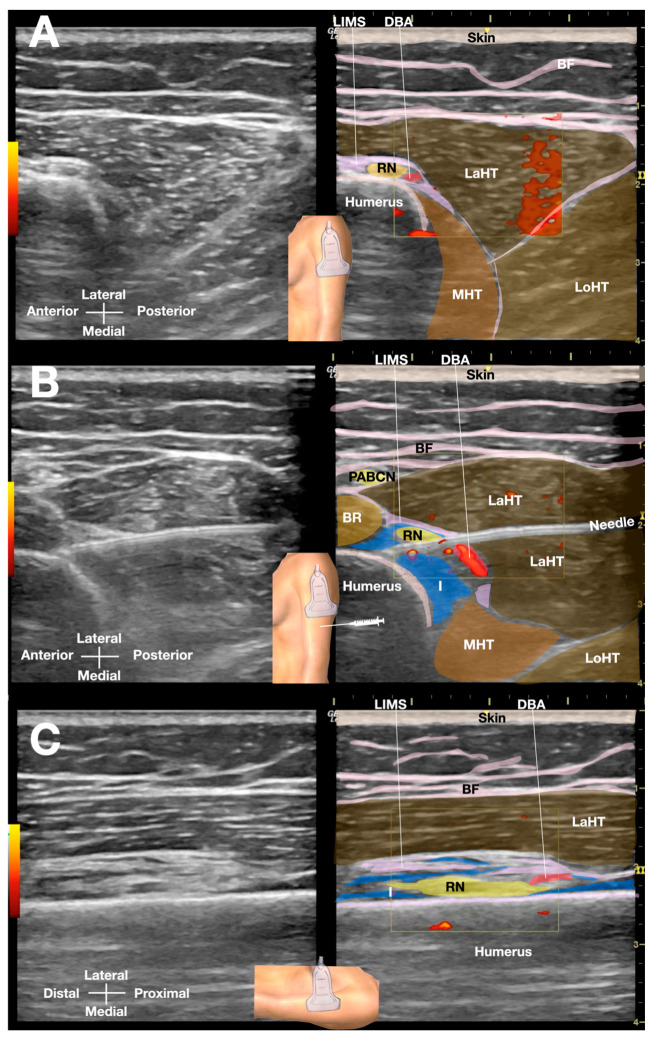
Ultrasound-guided hydrodissection of the radial nerve at the spiral groove. (**A**) Short-axis view showing the pre-hydrodissection sonoanatomy of the radial nerve in the spiral groove, visualizing the radial nerve adjacent to the humeral cortex and within the lateral intermuscular septum. (**B**) Post-hydrodissection appearance of the radial nerve, completely separated from the humerus, lateral intermuscular septum, and lateral head of the triceps by the injectate (5% dextrose in water without local anesthetics). (**C**) Long-axis view of the radial nerve in the spiral groove post-hydrodissection, with the injectate separating the nerve from the bony cortex of the humerus and the surrounding soft tissues. BF: brachial fascia; DBA: deep brachial artery; I: injectate; LaHT: lateral head of triceps; LIMS: lateral intermuscular septum; LoHT: long head of triceps; MHT, medial head of triceps; RN: radial nerve.

The patient was positioned side-lying for the procedure, targeting the proximal spiral groove using a posterior-to-anterior in-plane needle approach. After numbing the skin with 1% lidocaine, a total of 50 mL of D5W with no LA was injected under real-time sonographic guidance to hydrodissect the radial nerve in the spiral groove (Figure 1 and Video S1). Immediate post-procedural assessment revealed marked improvement in wrist extension, indicating a positive therapeutic response (Figure S3 and Video S2).

It is noteworthy that MRI was not performed due to the classic clinical presentation of compressive neuropathy (acute wrist drop without trauma), supported by ultrasound findings. The patient’s rapid response to hydrodissection further obviated the need for advanced imaging.

In a follow-up telephone interview conducted one month after the procedure, the patient reported significant improvement in wrist drop and extensor pollicis longus (EPL) dysfunction following US-guided HD. At that time, she indicated no residual impairment in wrist function and described complete restoration of normal daily activities. During a three-month follow-up telephone interview, the patient reported continued full restoration of left wrist function and strength. She noted no disturbances to her daily activities due to the episode of wrist drop from radial nerve palsy.

## 3. Discussion

Radial nerve palsy, most commonly resulting from compression at the spiral groove, also known as “Saturday night palsy” [15], accounts for 15–20% of peripheral nerve injuries [4], with a well-documented male predominance (70.1%) and frequent association with trauma (e.g., humeral fractures) or systemic conditions such as diabetes [5,16,17]. The underlying pathophysiology ranges from reversible neurapraxia [18] to severe neurotmesis [19], with prognosis critically dependent on the degree of nerve injury [18,19,20]. Clinically, patients present with the classic triad of wrist drop, finger extension weakness, and preserved elbow extension, while sensory deficits, when present, localize to the dorsal hand [5,21]. Although electrodiagnostic studies (nerve conduction velocity tests and electromyography) remain the gold standard for evaluation, high-resolution ultrasound has emerged as a valuable diagnostic tool, demonstrating 89% sensitivity in detecting compressive lesions through dynamic assessment of nerve gliding, nerve swelling, fascicular disruption, or perineural fibrosis [6,22,23,24]. The most common radial nerve neuropathy occurs at the spiral groove, colloquially known as “Saturday night palsy” or “honeymooner’s palsy” due to the prolonged external compression of the arm. Symptoms include weakness of wrist and finger extensors and sensory impairment in the dorsal arm and hand. However, in our case, pure motor symptoms occurred without sensory deficits, and the typical symptom was wrist drop. The absence of sensory deficits in this case could suggest selective compression of the motor fibers of the radial nerve or sparing of the superficial sensory branch. However, the rapid resolution of motor symptoms after hydrodissection at the spiral groove supports compression at this site, where the nerve is vulnerable to extrinsic pressure. While deep branch entrapment (e.g., radial tunnel syndrome) typically presents with lateral elbow pain and motor weakness, our patient’s clinical and sonographic findings localized the pathology to the spiral groove.

### 3.1. Current Management Strategies and Their Limitations

The standard approach to non-traumatic radial nerve palsy remains conservative management, including wrist splinting and nerve gliding exercises, though recovery typically requires 3–6 months and may take as long as 68 months [5,15,21,25,26,27,28]. This prolonged recovery period imposes significant functional limitations, economic burdens due to lost productivity, and psychological strain on patients. Surgical interventions, such as neurolysis or tendon transfers, are typically reserved for refractory cases or open injuries, yet these procedures carry inherent risks, including infection, scarring, and variable functional outcomes [5,29,30].

Recent studies have explored ultrasound-guided hydrodissection (HD) as a minimally invasive alternative. For example, Chen et al. (2018) reported complete motor and sensory recovery in a case of chronic compressive radial nerve palsy following two sessions of HD with 5% dextrose in water (D5W) [8]. Similarly, Su et al. (2020) utilized D5W with shear wave elastography to treat post-surgical radial nerve entrapment due to fibrosis, demonstrating significant functional improvement [6]. Despite these promising results, robust randomized controlled trials (RCTs) evaluating HD in acute radial nerve palsy remain lacking. To contextualize existing evidence, Table 1 systematically summarizes published studies on HD for radial neuropathy, comparing therapeutic approaches (e.g., D5W, saline, corticosteroids, platelet-rich plasma), clinical presentations (acute vs. chronic), and functional outcomes. Notably, studies addressing superficial radial nerve injury (purely sensory deficits) were excluded from this review.

### 3.2. Novelty and Unique Clinical Implications of This Report

This manuscript presents the first documented case of acute radial nerve palsy successfully treated with ultrasound-guided HD using D5W without local anesthetics (LAs), resulting in immediate functional restoration. This case highlights three critical advances in the management of compressive neuropathies. First, HD played a dual diagnostic and therapeutic role: unlike electrodiagnostic studies, which require a 2–3-week delay to assess injury severity, immediate post-procedural functional recovery confirmed neurapraxia as the primary pathology, eliminating diagnostic uncertainty [31]. Second, this approach represents a paradigm shift in treatment strategy, as it circumvents the prolonged disability associated with traditional conservative management, potentially reducing socioeconomic burdens linked to lost work capacity and prolonged rehabilitation. Third, the advantage of utilizing D5W without LA lies in its capacity to facilitate immediate reversal of compressive neurapraxic neuropathy. This approach enables accurate assessment of the mechanical decompression effect achieved through hydrodissection, unobscured by the transient neural blockade that would result from local anesthetic administration.

Prognosis depends on injury severity: neurapraxia typically resolves within 2–3 months, whereas Sunderland grade IV/V injuries or denervation on EMG portend poorer outcomes [5,32]. Future research should prioritize RCTs comparing early HD to conservative care, standardized injectate protocols, and the role of adjuvant therapies like platelet-rich plasma [33,34].

**Table 1:** Summary of studies on hydrodissection for radial neuropathy with motor deficits. Summary of key studies investigating ultrasound-guided hydrodissection (HD) for radial nerve pathology. The table compares study designs, *n* (numbers of patients), pathologies of the radial nerve, intervention, outcome measures, key findings, and limitations across published reports.

Study (Year) [Citation]	Design	*n*	Pathologies of the Radial Nerve	Intervention	Outcome Measures	Key Findings	Limitations
Gill et al. (2022) [9]	Case Series	11	Radial tunnel syndrome (between the two supinators, not in the spiral groove) with average duration of 24.3 months	US-guided HD of PIN (saline + corticosteroid)	VAS	100% symptom resolution, 4 patients needed HD only once, 4 needed twice, and 2 needed three times	Small sample; no control group
Chen et al. (2018) [8]	Case Report	1	Chronic compressive radial nerve palsy (2 months of failed conservative care)	Monthly US-guided HD (D5W × 2 sessions)	VAS, muscle power, nerve conduction, needle electromyography studies	Nearly full motor/sensory recovery 3 weeks after the second HD. Complete resolution of her symptoms 2 months after second HD	Single case; no functional scores
García de Cortázar et al. (2018) [33]	Case Report	1	Radial nerve section (post-traumatic)	US-guided PRP injection	EMG, manual muscle testing	Full function recovery and EMG showed complete reinnervation at 14 months after injury and 11 months after PRP injection	Single post-trauma case
Su et al. (2020) [6]	Case Series	2	Radial nerve entrapment in the spiral groove (post-surgical fibrosis and scarring)	US-guided HD (D5W) to the fibrotic and scarring area detected by SWE	VAS, muscle power, shear wave velocity of the radial nerve, neurodiagnostic studies (i.e., nerve conduction velocity and electromyography) two months after the surgery and after HD	Significant improvement in VAS immediately after D5W HD Significant improvement in muscle power, neurodiagnostic studies also showed significant improvement Erythema, allodynia disappeared	Small sample; heterogeneous presentation of the two patients

Abbreviations: D5W = 5% dextrose in water; PRP = platelet-rich plasma; SWE = shear wave elastography; US = ultrasound; VAS = visual analog scale; MRC = Medical Research Council; EMG = electromyography.

Beyond the radial nerve, research indicates that HD can benefit other entrapment neuropathies. In carpal tunnel syndrome (median nerve compression), randomized trials have demonstrated that perineural injection of 5% dextrose yields greater symptom improvement and reduction in median nerve swelling than control treatments [7,10,11,12,13,35,36,37,38,39,40,41,42,43]. Similarly, case reports have documented successful US-guided HD in ulnar nerve entrapment at the elbow and in other nerve compression syndromes [9,44,45,46,47]. Collectively, these findings suggest that perineural HD can effectively alleviate nerve compression and improve function in select patients, potentially serving as a surgery-sparing alternative in the management of compressive neuropathies [8,9,36].

### 3.3. Limitations and Future Directions

Despite the favorable outcome, this report has several limitations. The most significant limitation is the absence of electrodiagnostic studies [13,20,48,49,50,51] (nerve conduction studies and electromyography). While ultrasound supported the diagnosis of a focal radial neuropathy, electrodiagnostic testing is crucial for confirming the diagnosis, localizing the site of compression, and assessing the severity and type of nerve injury. Without these studies, we could not definitively rule out other potential causes of wrist drop or objectively quantify the degree of nerve recovery. Although ultrasound supported the diagnosis of a focal radial neuropathy and electromyography has limited sensitivity in the first 2–3 weeks of an acute nerve injury [15], the absence of nerve conduction data means that our assessment of improvement relied solely on clinical examination and ultrasound findings.

Second, as this is an isolated case without a control group, we cannot definitively exclude the possibility of spontaneous recovery or the placebo effect. The timing of the recovery—occurring immediately after the HD—strongly suggests a true therapeutic benefit, and importantly, no neural blockade medication was used (avoiding the confounder of a temporary anesthetic effect). Nonetheless, one case cannot prove causation, and the natural history of acute compressive radial palsy is variable.

Finally, the generalizability of this observation is limited. A single successful outcome does not guarantee that US-guided HD will work for all cases of radial nerve palsy; factors such as the severity of nerve injury (neurapraxia vs. axonotmesis), chronicity, or anatomical variations could influence efficacy. While generally considered safe, US-guided HD carries potential risks, including infection, bleeding, nerve injury, and an allergic reaction to the injectate. These risks can be minimized through careful hydrodissection techniques, real-time ultrasound guidance, and the absence of neural blocking medication, as demonstrated in this case.

Further research—ideally in the form of larger case series or controlled trials—is needed to validate the efficacy of US-guided HD, optimize the technique (e.g., timing, injectate volume, with or without LA), and identify which patients with compressive radial neuropathy are most likely to benefit from this approach.

### 3.4. Lessons Learned from This Case Report

This case underscores several key insights for clinicians managing acute compressive radial neuropathy:

- **Early Intervention:** This case suggests that early intervention with US-guided HD in acute compressive radial nerve palsy may lead to rapid and complete recovery, potentially avoiding the prolonged disability associated with conservative management. Additionally, it diagnostically supports the primary pathology of neurapraxia, which is largely reversible.
- **Importance of Ultrasound:** High-resolution ultrasound is valuable for both diagnosing and guiding treatment of peripheral nerve entrapments like radial nerve palsy.
- **D5W without LA as a Safe Injectate:** D5W without LA appears to be a safe and effective injectate for hydrodissection, minimizing the risk of nerve block and allowing for assessment of the true endpoints in neural decompression.
- **Need for Further Research:** While this case is promising, it highlights the need for further research, including controlled trials and electrodiagnostic confirmation, to validate the efficacy of US-guided HD and identify appropriate candidates for this intervention.

## 4. Conclusions

This case report presents a novel therapeutic approach for acute radial nerve palsy, demonstrating successful and immediate neurological recovery through ultrasound-guided hydrodissection (HD) using 5% dextrose water (D5W) without local anesthetics. Our findings illustrate the potential of this minimally invasive technique to achieve real-time peripheral nerve decompression while simultaneously serving as a diagnostic tool to confirm the presence of reversible neurapraxia. The case provides compelling clinical evidence that early ultrasound evaluation followed by guided HD with this benign injectate can produce both immediate and sustained neurological improvement in compressive neuropathies.

The clinical implications of these findings are potentially significant, suggesting a paradigm shift in the management of acute compressive neuropathies. By offering the possibility of rapid functional recovery, this approach may substantially reduce the disability period typically associated with conservative management strategies. However, to establish this technique as a validated treatment option, several critical research priorities must be addressed through rigorous scientific investigation.

Future studies should employ controlled designs with larger patient cohorts to objectively evaluate the efficacy of ultrasound-guided HD for radial nerve palsy. Such research should focus on optimizing technical parameters including injection timing, volume, and frequency, while incorporating electrodiagnostic confirmation of nerve recovery patterns. Additionally, careful attention must be paid to developing evidence-based patient selection criteria, with particular consideration given to symptom duration, nerve injury severity (differentiating neurapraxia from axonotmesis), and the presence of comorbid conditions such as diabetes mellitus that may affect nerve recovery.

Until more comprehensive clinical data become available, ultrasound-guided HD with D5W should be regarded as an investigational intervention for compressive radial neuropathy. Nevertheless, this case provides important preliminary evidence that merits serious consideration in both clinical and research contexts. The technique’s potential to offer immediate diagnostic and therapeutic benefits while avoiding the limitations of traditional management approaches makes it a promising avenue for future investigation in peripheral nerve disorders.

## Data Availability

Data is included in the manuscript.

## Supplementary Materials

The following supporting information can be downloaded at https://www.mdpi.com/article/10.3390/diagnostics15151880/s1, Figure S1: Clinical presentation of the patient’s left-hand radial nerve palsy. Video S1: Video of clinical presentation of patient’s left-hand radial nerve palsy. Figure S2: Plain radiographic evaluation of the upper extremity and cervical spine. Figure S3: Clinical improvement following ultrasound-guided hydrodissection with dextrose 5% in water without local anesthetic. Video S2: Video of post-hydrodissection assessment of a patient with acute radial nerve palsy. File S1: GAMER Checklist. Reference [52] is cited in the supplementary materials.
