# Supplementary material for: Confirming the Presence of Neurapraxia and Its Potential for Immediate Reversal by Novel Diagnostic and Therapeutic Ultrasound-Guided Hydrodissection Using 5% Dextrose in Water Without Local Anesthetics: Application in a Case of Acute Radial Nerve Palsy
Source: Diagnostics (Basel). 2025 Jul 26;15(15):1880. doi: 10.3390/diagnostics15151880 (PMC12346177; doi:10.3390/diagnostics15151880)
Supplement: Supplementary file 1 [file diagnostics-15-01880-s001.zip › diagnostics-3691803-supplementary.pdf]

diagnostics

Supplemetary materials:

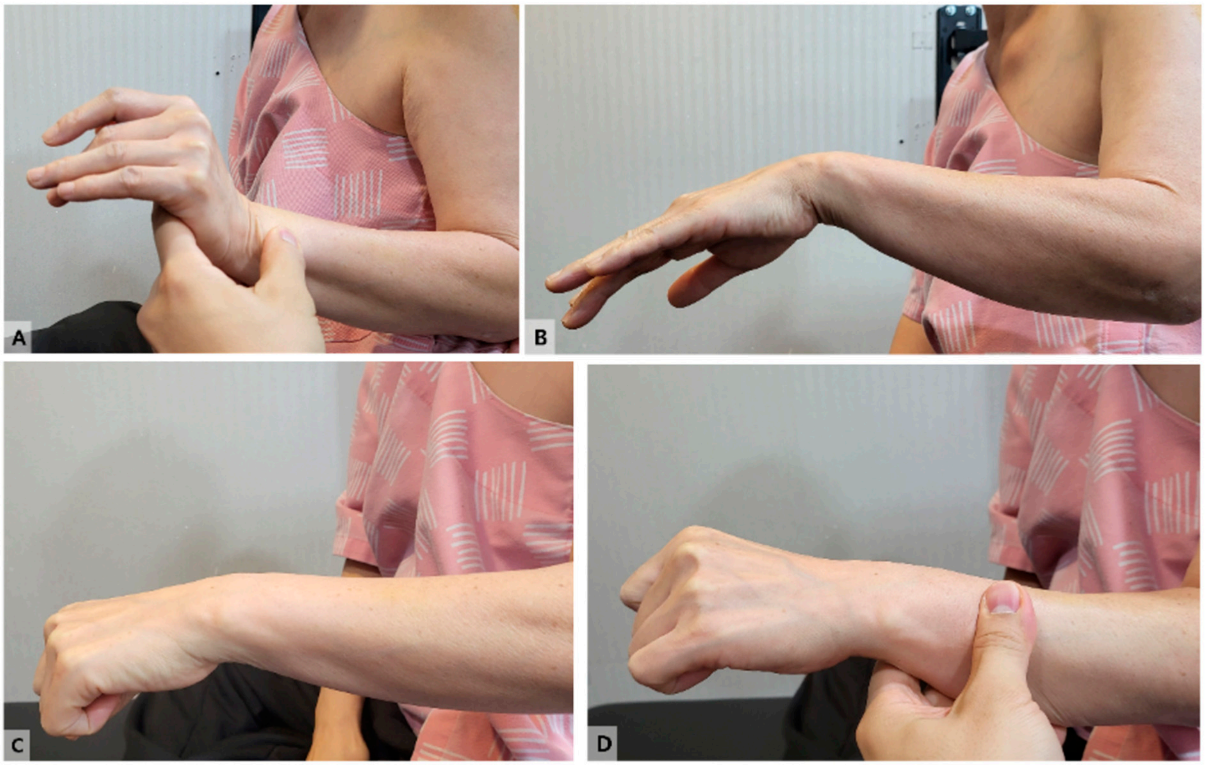

Supplementary Figure S1. Clinical presentation of the patient's left-hand radial nerve palsy. A) Preserved passive Range of motion of the left wrist. B) Pronounced wrist drop observed in the resting position. C-D) Markedly limited active wrist extension.

Supplementary Video S1.

https://www.dropbox.com/scl/fi/l1ehd6jexyr4pgpnzh5rr/radial-nerve-palsy-PE-combined.mp4?rlkey=w0xiqlpr8i85te1pdev5wqlkd&dl=0

Supplementary Video S1 Title: Video of Clinical Presentation of Patient's Left-Hand Radial Nerve Palsy

Supplementary Video S1 Legend: This video demonstrates that the patient has preserved passive range of motion in the left wrist, pronounced wrist drop observed in the resting position, and marked weakness in active left wrist and thumb extension.

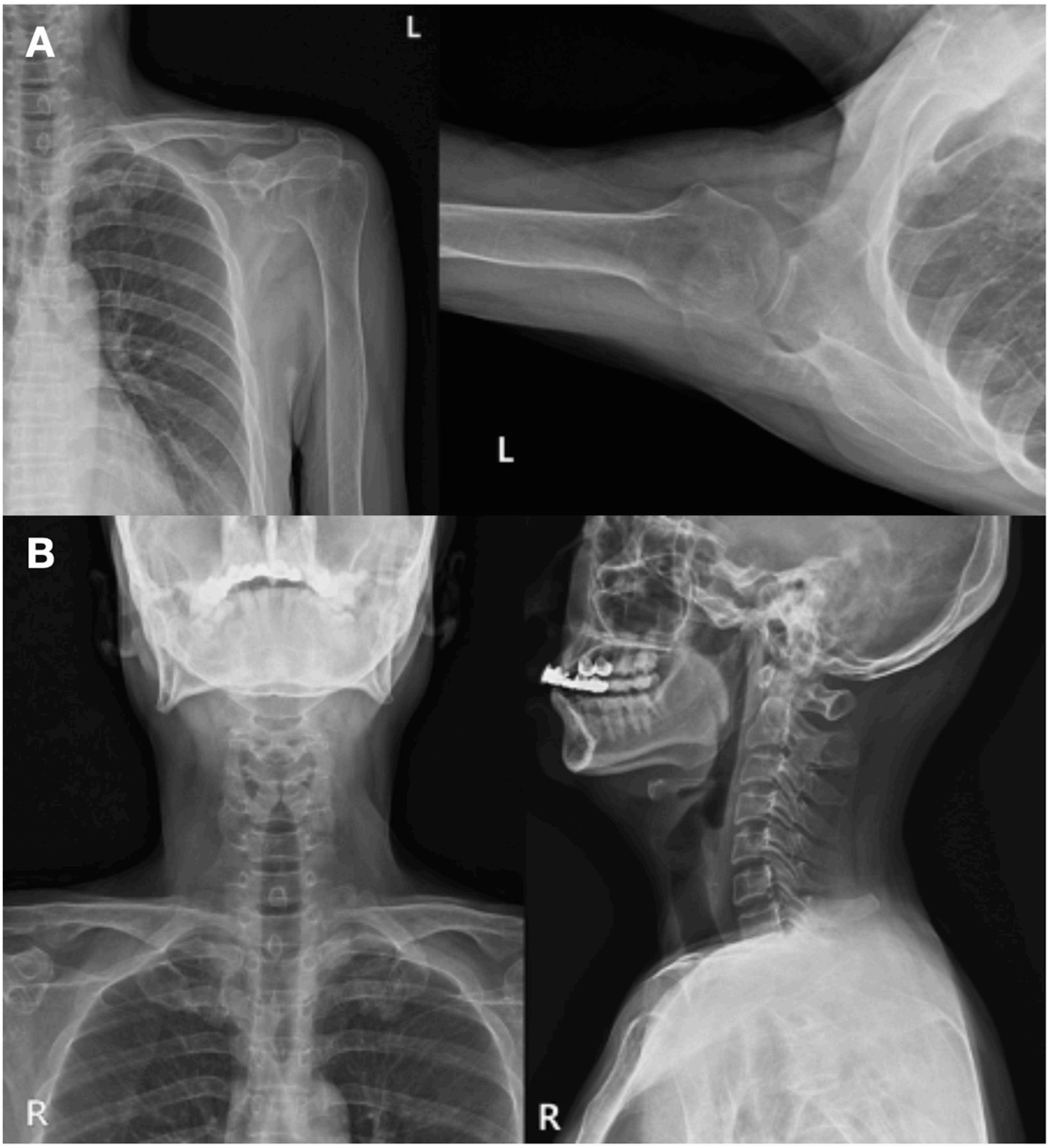

Supplementary Figure S2. Plain radiographic evaluation of the upper extremity and cervical spine. A) Shoulder radiographs (true anteroposterior and superior-inferior axial views) show no evidence of fractures, dislocations, or other structural abnormalities. B) Cervical spine radiographs (anteroposterior and lateral views) demonstrate no bony lesions or cervical pathology.

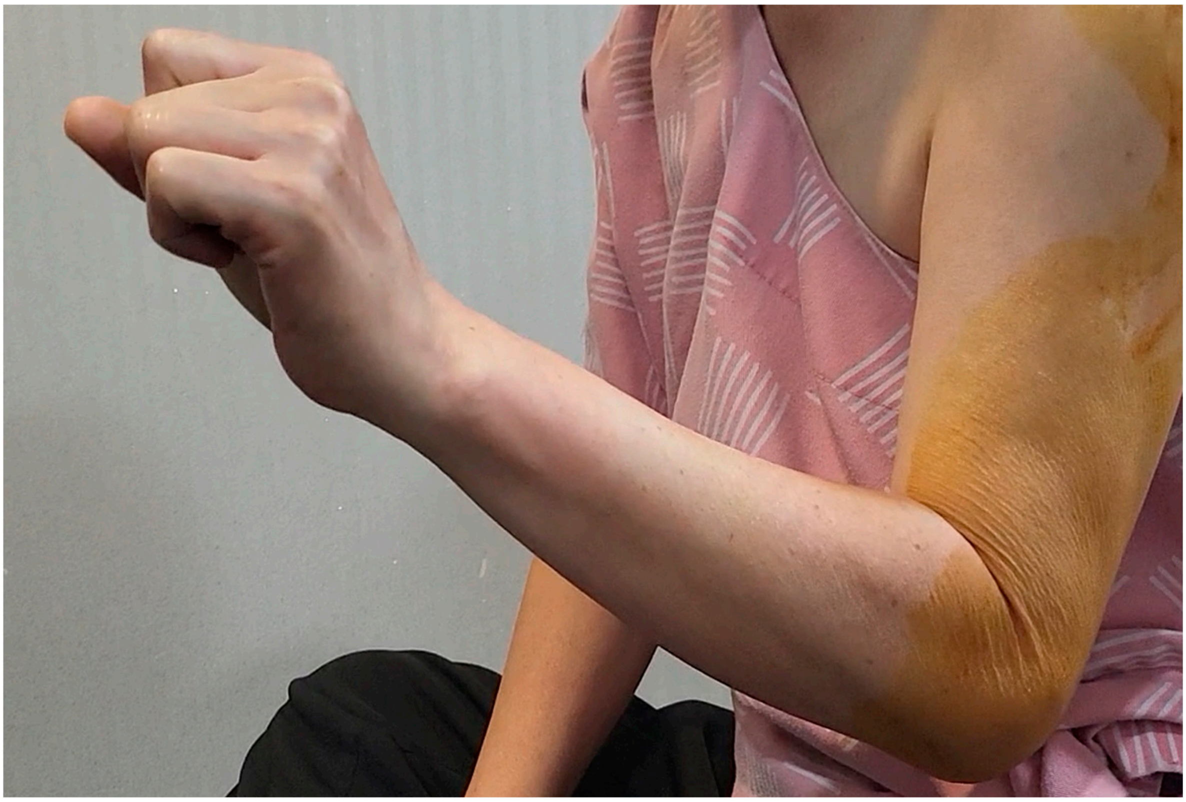

Supplementary FigureS3. Clinical improvement following Ultrasound-guided hydrodissection with dextrose 5% in water without local anesthetic. Recovery of active wrist extension immediately after the procedure, demonstrating resolution of wrist drop.

<<<<Supplementary Video S2>>>>
[*https://www.dropbox.com/scl/fi/bfknznmqs4v53vsj34qsd/Post-procedure-Assessment.mp4?rlkey=k0l1akw6wmgqn2ydsv8ii8qrw&dl=0*](https://www.dropbox.com/scl/fi/bfknznmqs4v53vsj34qsd/Post-procedure-Assessment.mp4?rlkey=k0l1akw6wmgqn2ydsv8ii8qrw&dl=0)

Supplementary Video S2 Title: *Post-Hydrodissection Assessment of a Patient with Acute Radial Nerve Palsy*

Supplementary Video S2 Legend: *This video showcases the clinical improvement following ultrasound-guided hydrodissection using 5% dextrose in water without local anesthetic. It highlights the recovery of active wrist extension immediately after the procedure, demonstrating the resolution of wrist drop.*

File S1

We confirm that no AI tools were used to generate or alter the scientific content, hypotheses, or conclusions of this manuscript. Online grammar-checking tools (Free Grammar Checker (Online Editor), https://www.grammarcheck.net › editor) were solely used for language polishing of pre-written text. To ensure full transparency, we have completed the **GAMER checklist** and included it as **File S1 [1]**.

Key disclosures:

1. **AI Usage**: Only for post-draft language editing (no content generation/analysis).
2. **Human Oversight**: All AI-polished text was verified by authors for accuracy.
3. **Originality**: All data, figures, and conclusions are human-derived.

GAMER Checklist

GAMER Items	Our Compliance
**AI in Drafting**	No AI used for content generation or data interpretation
**AI in Editing**	Grammar/ spelling checks only (Free Grammar Checker (Online Editor), https://www.grammarcheck.net › editor)
**Human Verification**	All edits reviewed by authors for scientific accuracy
**Bias/Originality Checks**	Manual plagiarism screening; no AI text generators employed

References

1. Luo, X., et al., *Reporting guideline for the use of Generative Artificial intelligence tools in MEdical Research: the GAMER Statement.* BMJ Evid Based Med, 2025.
